# Prophylactic use of laxative for constipation in critically ill patients

**DOI:** 10.4103/1817-1737.69113

**Published:** 2010

**Authors:** Yasser Masri, Jawed Abubaker, Raees Ahmed

**Affiliations:** *Department of Critical Care Medicine, Surgical Intensive Care Unit, Dubai, United Arab Emirates*; 1 *Medical Intensive Care Unit, Rashid Hospital Trauma Center, Dubai, United Arab Emirates*

**Keywords:** Constipation, critically ill patients, prophylaxis

## Abstract

**BACKGROUND:**

This study was designed to evaluate the use of laxative prophylaxis for constipation in intensive care unit (ICU) and the impact of early versus late bowel movement on patient’s outcome.

**METHODS:**

The study was a prospective, randomized controlled trial in critically ill ventilated adult patients, who were expected to stay on ventilator for >72 h. Control group did not receive any intervention for bowel movement for the first 72 h, whereas interventional group received prophylactic dose of lactulose 20 cc enterally every 12 h for the first 72 h. The parameters measured during the study were admission diagnosis, age, gender, comorbid conditions, admission Simplified Acute Physiologic Score (SAPS II), sedative and narcotic agents with doses and duration, timing and tolerance of nutrition, daily assessment of bowel movement, total use of prokinetic, doses of suppositories, and enema for first bowel movement, total number of days on ventilator, weaning failures, extubation or tracheostomy, ICU length of stay, and death or discharge.

**RESULTS:**

A total of 100 patients were enrolled, 50 patients in each control and interventional group. Mean age was 38.8 years, and both groups had male predominance. Mean SAPS II score for both was 35. Mean dose of Fentanyl (323.8 ± 108.89 mcg/h in control and 345.83 ± 94.43 mcg/h in interventional group) and mean dose of Midazolam (11.1 ± 4.04 mg/h in control and 12.4 ± 3.19 mg/h in interventional group). There were only two (4%) patients in control, while nine (18%) patients in interventional group who had bowel movement in <72 h (*P* < 0.05). Mean ventilator days were 16.19, and 17.36 days in control and interventional groups, respectively. Subgroup analysis showed that the patients who moved bowel in <5 days in both groups had mean ventilator days of 18.5, whereas it was 15.88 days for the patients who moved bowel after 5 days in both groups (*P*< 0.05). Mean ICU days for control was 21.15 ± 10.44 and 20.77 ± 8.33 days for interventional group. Forty-eight (96%) patients in each group were discharged from the ICU. Two (4%) patients died in ICU in each group.

**CONCLUSIONS:**

Laxative prophylaxis can be used successfully to prevent constipation in ICU patients. Late bowel movement >5 days is associated with less ventilator days, compared to early <5 days bowel movement.

American Gastroenterological Association published guidelines on constipation, which defines constipation as the frequency of feces evacuation of <3 times per week, feeling of incomplete rectal evacuation, hard stool, struggling to pass stools, and need to tap for rectal emptying.[1] Incidence of constipation in critically ill patients is reported to be as high as 83%,[2] at present there is no clear published guidelines or data available on prevention and treatment of this commonly occurring problem in critically ill patients.

Most of the data in the literature is on opioid-related constipation in cancer and terminally ill patients. Opioids are also very frequently used agents in intensive care units (ICUs), but there are several other factors, which can delay bowel movement in critically ill patients. Constipation may go unnoticed in critically ill patients, but may also cause symptoms including abdominal distension and pain, gastric fullness, nausea, vomiting, anorexia, confusion, and overflow diarrhea.[3]

The impact of constipation in critically ill patients is largely unknown, but observational studies have found a relation between a prolonged length of stay (LOS) and increased mortality.[2,4] In a multicentered study; Montejo prospectively investigated the frequency of nonhemorrhagic gastrointestinal complications in 400 ICU patients receiving enteral feeding. Almost two-thirds of subjects developed one or more gastrointestinal complications; high gastric residuals (39%) and constipation (15.7%) were most common. Patients with gastrointestinal complications had longer ICU stays (20.6 ± 1.2 days versus 15.2 ± 1.3 days) and higher mortality (31% versus 16%) compared to the group without gastrointestinal (GI) complications.[5]

## Methods

This study was conducted to evaluate the effect of laxative prophylaxis to prevent constipation in critically ill patients. The study was a prospective, randomized controlled trial in critically ill-ventilated adult patients admitted to the ICU of a teaching hospital. The study protocol was approved by research and ethical committee of the hospital. The need for written consent was exempted by the committee as the interventional drug was used routinely in the ICU, and the dose used for the study was safe.

Surgical ICU at Rashid Hospital is a 16-bed unit with mostly trauma and surgical patients. Total hospital beds capacity is 550 beds, and there is a separate 30-bed Medical ICU.

Data were collected between January and June 2009. All adult critically ill patients admitted to the unit were reviewed for eligibility. Inclusion criteria were adult patients admitted and ventilated who are expected to stay more than 3 days in the ICU and have no contraindication for enteral nutrition. The exclusion criteria were pediatrics patients, spontaneously breathing patients, mechanically ventilated patients who are expected to stay less than 3 days and patients who had major bowel surgery or any surgery which prevents the enteral feeding.

Patients enrolled in the study were randomized in a control group and intervention group. Randomization was done using sealed envelopes with the options inside the sealed envelope of intervention and control, available for each shift. The charge nurse of the shift randomly picked up the envelope, and the patient was assigned to “intervention” and “control group.”

Control group did not receive any laxative, prokinetic agent, or enema for bowel movement for the first 3 days of ICU admission. Intervention group received lactulose empirically for the first 3 days of ICU admission. The dose of lactulose was 20 mL (approximately 13 g) twice a day (every 12 h), started within 4–6 h of ICU admission. The production of stool was scored as present if the nurse estimates the volume as >100 mL. After completion of 3 days, the management of constipation in both the groups was left on treating physician discretion. All patients were fed enterally as per ICU nutrition protocol.

The parameters measured during the study were admission diagnosis, age, gender, comorbid conditions, admission Simplified Acute Physiologic Score (SAPS II), sedative and narcotic agents with doses and duration, timing and tolerance of nutrition, daily assessment of bowel movement, total use of prokinetic (metoclopromide 10 mg intravenous), and total glycerin suppositories and phosphate enemas used for the first bowel movement, total number of days on ventilator, weaning failures, extubation or tracheostomy, ICU length of stay, and death or discharge.

The primary endpoint of the study was timing of first bowel movement (<72 h) in both the groups to see the effect of lactulose for the prophylaxis of constipation. Secondary endpoints were day of first bowel movement and the impact of early versus late bowel movement on number of days on ventilator, length of ICU stay, weaning, extubation, tracheostomies, and ICU mortality.

The control and intervention groups were analyzed for statistical significance (*P* < 0.05) by Chi-square test or Fisher exact test, or Student *t*-test. The analysis was performed by SPSS Statistics 17.0.1 (SPSS, Chicago, IL).

## Results

Over 6 months period 251 patients were admitted to the ICU. About 100 patients met the inclusion criteria and were enrolled, 151 patients were not included due to blunt abdominal injury or postoperative bowel resection. Fifty patients were randomized in each control and interventional groups. Mean age was 38.8 in control and 37.5 years in interventional group. Both groups had predominance of male patients (78% in control and 81% in intervention group). Most common diagnosis in both groups was polytrauma (62% in control and 65% in intervention group) followed by sepsis (15% in control and 18% in intervention group). Mean SAPS II score for both the groups was 35. All (100%) patients were ventilated. The most commonly used analgesic and sedative agents were Fentanyl and Midazolam, with mean dose of fentanyl (323.8 ± 108.89 mcg/h in control and 345.83 ± 94.43 mcg/h in interventional group). The mean dose of midazolam used for both the groups was (11.1 ± 4.04 mg/h in control and 12.4 ± 3.19 mg/h in interventional group). Average duration of sedation and analgesia in both control and interventional groups was 5 ± 2 days.

There was no contraindication for feeding in both the groups. All patients were fed with isoosmolar enteral feeding (Osmolite; Ross) through the nasogastric tube. Timing of enteral feeding in the control group showed 72% of patients were fed within 24 h and 25% within 24–48 h. In intervention group, 75% were fed within 24 h and 22% within 24–48 h. The average rate of feeding in both the groups was 62 mL/h per day. Two (4%) patients in control group and nine (18%) patients in interventional group moved their bowel in <72 h (*P* < 0.05).

After 72 h, the doses of laxative, prokinetic agents, suppositories and enema used in both the groups to induce first bowel movement were not significantly different. By seventh day of the study, 100% of patients in both groups had their first bowel movement. Almost 50% of patients in both groups moved their bowel by fifth day (46% patients in control and 58% patients in interventional groups).

When ventilators days were reviewed and compared between the two groups, there was no significant difference in number of ventilator days for the entire control and intervention groups (mean ventilator days were 16.19 and 17.36 days). Table 1 shows subgroup analysis of patients who moved their bowel within 72 h in both control and intervention groups, which also did not show any difference in mean ventilator days. However when groups were divided into ≤5 days bowel movement and >5 days bowel movement, we found that the patients who moved bowel in ≤5 days in both control and intervention groups had mean ventilator days of 18.5, where it was 15.88 days for the patients who moved bowel after 5 days in both groups (*P* < 0.05).

**Table 1:** Comparison of ventilator days between control and intervention groups

Mean days on mechanical ventilation	Control group	Intervention group
Mean days on ventilator for the entire group	16.19	17.36
Mean days on ventilator for bowel movement <72 h	17.21	17.92
Mean days on ventilator for bowel movement >72 h	16.43	16.23
Mean days on ventilator for bowel movement <5 days	18.39	8.61
Mean days on ventilator for bowel movement >5 days	15.98	15.78

Mean ICU days was 21.15 ± 10.44 for control group and 20.77 ± 8.33 days for interventional group. More than 50% patients in both the groups were successfully extubated during their ICU stay. Total of 23 (46%) patients in control groups and 21 (42%) in intervention group had tracheostomy done. All patients who underwent tracheostomy in both groups were successfully liberated from the ventilator before their ICU discharge. Two (4%) patients died in each group, whereas 48 (96%) patients in each group were successfully discharged from the ICU [Table 2].

**Table 2:** Comparison of results between control and interventional groups

	Control group	Objective asthma control
Total number	50	50
Mean age	38.2	37.5
Male, %	78	81
Female, %	22	19
Mean SAPS II	35	35
Mean dose fentanyl	323.8 ± 108.89 mcg/h	345.83 ± 94.43 mcg/h
Mean dose midazolam	11.1± 4.04 mg/h	12.4 ± 3.19 mg/h
Enteral nutrition
Started within 24 h	72%	75%
Started between 24 and 48 h	25%	22%
Average infusion rate	62 cc/ h/day	62 cc/h/day
Bowel movement in <72 h*	2 (4) patients	9 (18) patients
Suppositories and enema mean doses	3.55	3.43
Mean ICU stay	21.15 ± 10.44 days	20.77 ± 8.33 days
Tracheostomy	23 (46) patients	21 (42)
Death	2 (4) patients	2 (4) patients
Discharged	48 (96) patients	48(96) patients

SAPS II = Simplified acute physiologic score II; ICU = Intensive care unit

* *P* < 0.05, Figures in parentheses are in percentage

## Discussion

Constipation is one of the most common findings in ICU patients. The causes are multiple and can range from simple immobility to fatal bowel obstruction. At present, there are no guidelines or recommendations available to manage constipation in ICU. To our knowledge, this is the first randomized controlled trial in critically ill patients, in which empiric laxative was used on admission day to induce early bowel movement. We additionally analyzed impact of early versus late bowel movement on days on ventilator, length of ICU stay, weaning failure, tracheostomies, and ICU mortality.

Commonly used laxatives in the ICU are lactulose and polyethylene glycol (PEG). The most use of these agents in the ICU is either on nursing request or demand as per physician orders. Lactulose and PEG are both osmotic laxatives. Recently, Van der Spoel *et al*.[4] in their two-center randomized, double-blind, placebo-controlled study found that in critically ill patients, both lactulose and PEG are equally effective in promoting defecation than placebo. The lactulose solution which was used in their study was prepared by mixing 13 g of lactulose in 100 mL of sterile water given every 8 h. We used 20 mL of lactulose solution (approximately 13 g) every 12 h for our study group.

The reason we chose lactulose for our study group was for to its effectiveness, availability, cost, and safety profile. We found that prophylactic use of lactulose was associated with 18% bowel movement within 72 h in interventional group, compare to only 4% in control group. Opioid-induced constipation is a known entity in critically ill patients, and there are some studies in which narcotic antagonists medication such as naloxone and methylnaltrexone, have been used to reverse and treat opioid-induced constipation. These studies confirmed the safety of narcotic antagonists which were used orally, subcutaneously, and intravenously for the reversal of opioid-induced constipation in terminally ill and critically ill patients.[6–9]

We did not specifically look into the incidence of opioid-induced constipation in our study group, however due to the fact that we used fentanyl for our patients, which belongs to the short acting narcotic class; the incidence must be insignificant.[10] The doses of narcotic agents used in both control and intervention groups were also similar and not statistically significant, which may supports our finding that early bowel movement <72 h in the intervention group was probably due to the laxative effect of lactulose prophylaxis.

Impact of constipation on outcome in critically ill patients, has not been extensively studied; however, there are some observational studies, one of them was published by Mostafa *et al*., in which they observed a statistically significant relationship between weaning failure from mechanical ventilation and constipation.[2] Later, in 2006, Van der Spoel *et al*.[4] confirmed the findings of the Mostafa’s study and showed an increase in the duration of mechanical ventilation among patients who remained constipated for more than 6 days in the ICU. Moreover, his study also showed shorter LOS in ICU among patients who had early bowel movement. The most recently published data are from the observational study conducted in Brazil, published in 2009 by Nassar *et al*. The author studied 106 patients and found that the incidence of constipation in this cohort was 69.9%. In his study, constipation was not related to any of the prognostic variables studied, namely renal replacement therapy, days free of mechanical ventilation, length of ICU stay, ICU mortality, and hospital mortality.[11]

We statistically analyzed the number of days on ventilator in our study population; we found that when the entire control and intervention group was compared, the mean number of days on ventilator was 16.19 and 17.36 days, respectively, which did not reach statistical significant. The mean numbers of days on ventilator were also statistically insignificant for the group with bowel movement <72 h and >72 h in both control and interventional groups. However when the mean ventilator days for the subgroup of patients who had bowel movement ≤5 days (18.5 days) and >5 days (15.88 days) in control and intervention groups were compared, the group with bowel movement >5 days had statistically significant less days on ventilator (*P* < 0.05).

The mean ICU length of stay was not significantly different when entire control and intervention groups, and subgroup analysis were compared (mean ICU days for control group was 21.15 ± 10.44 and 20.77 ± 8.33 days for interventional group). Unlike the finding published by Mustafa *et al*., we could not find any significant difference in weaning failure in our studied groups. More than 50% of our patients in both the groups were successfully extubated. Twenty-three (46%) patients in control and 21(42%) in intervention groups had tracheostomy. The most common indication for tracheostomy was low Glasgow Coma Scale and airway protection. There was no difference in ICU mortality, when the entire groups, group with bowel movement <72 h and groups with bowel movement ≤5 days and >5 days were compared.

The complications related to osmotic laxatives are mostly subjective, which includes fullness, distension, bloating, etc. In patients who are ventilated and sedated, it is almost impossible to collect the data on these subjective complications; however, 100% of our patients tolerated enteral nutrition, without any high residual or need for parenteral supplementation, which supports the finding that there were no major complications or side effects due to osmotic laxatives.

Our findings of this study supports that laxative prophylaxis can be safely and effectively used to initiate early bowel movement in critically ill patients requiring mechanical ventilation; however, unlike other published studies we could not find any positive impact of early bowel movement on any prognostic variables studied in our group population. In fact, our subgroup analysis showed that the early bowel movement had a negative impact on days on ventilation, with fewer ventilation days in-group with first bowel movement >5 days.

We feel that at present there are quite many challenges and unanswered questions on this important topic. First we may need to redefine constipation in critically ill patients, which is an entirely different population compared to constipated patients who belongs to other group of diseases. Secondly, what should be the optimal dose of lactulose for prevention of constipation as a prophylactic agent? We used twice-daily dose, which may be suboptimal and three times a day may be more effective. Last but not the least challenge is to confirm if timing early versus late bowel movement can impact the outcome in critically ill patients. Well-conducted high patient volume randomized control trials will be extremely helpful to answer these questions.

## Conclusions

Laxative prophylaxis with lactulose can be used successfully to prevent constipation in adult mechanically ventilated patients. Timing of first bowel movement may impact the number of days on ventilator. Late bowel movement >5 days is associated with less ventilator days, compared to early <5 days bowel movement.
